# Association of *MARC1*, *ADCY5,* and *BCO1* Variants with the Lipid Profile, Suggests an Additive Effect for Hypertriglyceridemia in Mexican Adult Men

**DOI:** 10.3390/ijms231911815

**Published:** 2022-10-05

**Authors:** Berenice Rivera-Paredez, Diana I. Aparicio-Bautista, Anna D. Argoty-Pantoja, Nelly Patiño, Jeny Flores Morales, Jorge Salmerón, Guadalupe León-Reyes, Rafael Velázquez-Cruz

**Affiliations:** 1 Research Center in Policies, Population and Health, School of Medicine, National Autonomous University of Mexico (UNAM), Mexico City 04510, Mexico; 2 National Institute of Genomic Medicine (INMEGEN), Mexico City 14610, Mexico; 3 Genomics of Bone Metabolism Laboratory, National Institute of Genomic Medicine (INMEGEN), Mexico City 14610, Mexico

**Keywords:** genetic risk score, *MARC1*, *ADCY5*, *BCO1*, polymorphism, dyslipidemia, Mexican population

## Abstract

Epidemiological studies have reported that the Mexican population is highly susceptible to dyslipidemia. The *MARC1, ADCY5*, and *BCO1* genes have recently been involved in lipidic abnormalities. This study aimed to analyze the association of single nucleotide polymorphisms (SNPs) rs2642438, rs56371916, and rs6564851 on *MARC1, ADCY5,* and *BCO1* genes, respectively, with the lipid profile in a cohort of Mexican adults. We included 1900 Mexican adults from the Health Workers Cohort Study. Demographic and clinical data were collected through a structured questionnaire and standardized procedures. Genotyping was performed using a predesigned TaqMan assay. A genetic risk score (GRS) was created on the basis of the three genetic variants. Associations analysis was estimated using linear and logistic regression. Our results showed that rs2642438-A and rs6564851-A alleles had a risk association for hypertriglyceridemia (OR = 1.57, *p* = 0.013; and OR = 1.33, *p* = 0.031, respectively), and rs56371916-C allele a trend for low HDL-c (OR = 1.27, *p* = 0.060) only in men. The GRS revealed a significant association for hypertriglyceridemia (OR = 2.23, *p* = 0.022). These findings provide evidence of an aggregate effect of the *MARC1, ADCY5,* and *BCO1* variants on the risk of hypertriglyceridemia in Mexican men. This knowledge could represent a tool for identifying at-risk males who might benefit from early interventions and avoid secondary metabolic traits.

## 1. Introduction

Dyslipidemias are characterized by an imbalance in the lipid circulating levels, such as cholesterol, low-density lipoprotein cholesterol (LDL-c), high-density lipoprotein cholesterol (HDL-c), and triglycerides [1]. Dyslipidemias are one of the main risk factors for metabolic diseases such as obesity, insulin resistance, and type 2 diabetes (T2D) in the Mexican population and at a global level [2]. Mexican adults are highly susceptible to developing metabolic disorders such as hypertriglyceridemia, whose prevalence is significantly higher in men (43.3%) than in women (23%) [3,4]. Both genetic and environmental factors contribute to dyslipidemias in varying degrees. It has been reported that specific risk alleles for hypertriglyceridemia have significantly higher frequency among Mexicans than in other populations [5]. Hence, many genetic studies employing single candidate genes and genome-wide association studies (GWAS) have provided compelling evidence that several gene variants are associated with dyslipidemias [6]. The single nucleotide polymorphism (SNP) rs2642438 on the *Mitochondrial Amidoxime Reducing Component 1 (MARC1)*, rs56371916 on *Adenylate cyclase 5 (ADCY5)*, and rs6564851 on the *β-Carotene-15,15’-oxygenase (BCO1)* genes have been recently related to lipid abnormalities [7,8,9].

The *MARC1* gene, which encodes the mitochondrial amidoxime-reducing component, has been involved in hepatic metabolic processes, e.g., activating *N*-hydroxylated prodrugs or reducing nitrite to produce nitric oxide [10]. The missense variant rs2642438 on the *MARC1* gene results in an alanine to threonine substitution at amino acid position 165 of the MARC1 protein (A165T) [11]. Recently, the rs2642438-A allele has been associated with higher triglyceride levels, lower HDL-c, and low liver enzyme levels [9,11]. It has been shown that *MARC1* deficiency can protect against cirrhosis [11] and decrease the severity of nonalcoholic fatty liver disease (NAFLD); however, the mechanism involved is unclear [9,12,13].

In mammals, cAMP is produced from ATP by a family of enzymes called adenylate cyclases (ADCYs). To date, 10 ADCY isoforms, coded by different genes, have been described in humans. These isoforms are differentially expressed across tissues and characterized by specific regulatory properties [14]. Adenylate cyclase 5 (ADCY5) is the isoform mainly expressed in neurons and myocardium [15] and may be the most abundant member of the ADCY family in human islets [16,17]. ADCY5 is a member of the membrane-bound ADCY family, which converts adenosine triphosphate (ATP) to the second messenger cyclic adenosine monophosphate (cAMP) and pyrophosphate [18]. The cAMP itself is a key regulator of glucose and lipid metabolism [19,20]. ADCY5 couples glucose to insulin secretion by converting glucose signals into cAMP production [21]. ADCY5 has been associated with cardiac complications such as congestive heart failure, fat distribution, insulin signaling, adipocyte function, and fatty acid oxidation [22,23]. It has been proposed that the rs56371916-C allele alters the binding affinity of sterol regulatory-element-binding protein 1 (SREBP1) and leads to differential *ADCY5* gene expression and cell-autonomous changes in fatty acid metabolism in mature adipocytes and differentiating osteoblasts [7]. Disruption of each, the regulator SREBP1, the variant rs56371916, and the target gene *ADCY5* could cause cellular changes as lipid oxidation relevant to high bone mineral density and T2D [7].

The *BCO1* gene is involved in carotenoid metabolism and has been recently linked with the development of coronary atherosclerosis and circulating cholesterol concentrations [24]. Specifically, the rs6564851 variant localized near the promoter region of the *BCO1* gene has been significantly associated with a reduction in total cholesterol levels and non–HDL-c in a cohort of young Mexican adults [25]. Recently, we reported that the rs656485-A allele is associated with a risk for hypertriglyceridemia only in the male group of middle-aged Mexican adults [8]. Therefore, the present study aims to investigate the lipid-linked variants in *MARC1*, *ADCY5*, and *BCO1* genes and test their aggregate effect on lipid profiles in Mexican adults belonging to the Health Workers Cohort Study (HWCS). These findings highlight the importance of continuing discovery and refinement of genetic variants that underlie the lipid abnormalities that are the most common cardiovascular risk factors in the Mexican population.

## 2. Results

### 2.1. Baseline Clinical Characteristics of the Study Population

A total of 1900 individuals were included in the study, of which 66.5% were women. The median age was significantly higher in women (52.3 years) than in men (46.3 years) (*p* < 0.001). The men group presented higher overweight, energy intake, and triglycerides than women (*p* < 0.05). While obesity, carbohydrate intake, and lipid metabolism parameters such as total cholesterol, HDL-c, and LDL-c levels were higher in women (*p* < 0.05) (Table 1). 

When demographics data were categorized by genotypes and sex for each SNP, male carriers of at least one rs2642438-A allele showed a higher prevalence of hypertriglyceridemia than noncarriers (GG: 55.6% vs. GA + AA: 65.7%; *p* = 0.028); to compare, women’s group showed lower total cholesterol levels than GG carriers (GG: 200 mg/dL vs. GA + AA: 196 mg/dL; *p*= 0.032). With respect to the rs56371916 SNP, only male carriers of at least one allele-C had lower total cholesterol (TT:194 mg/dL vs. TC + CC:187 mg/dL; *p* = 0.011) and HDL-c levels (TT:40.2 mg/dL vs. TC + CC: 38.8 mg/dL; *p* = 0.002) than noncarriers (Table S1).

### 2.2. Association Analyses between the rs2642438 on MARC1 and rs56371916 on ADCY5 with the Lipid Profile

We analyzed the association between the genetic variants and the lipid profile in the HWCS population by sex. We found a risk association between the rs2642438-A allele and high triglyceride levels only in the men group. This association was statistically significant under an additive (odd ratio (OR) = 1.57; 95% confidence interval (CI) = 1.10–2.24, *p* = 0.013) and dominant inheritance model (OR = 1.54; 95% CI = 1.04–2.28, *p* = 0.030). In addition, we found a borderline risk association between the rs56371916-C allele with HDL-c levels under an additive model (OR = 1.27, *p* = 0.060) (Table 2).

Consistently, in the linear regression analysis, we also observed a positive association between the rs2642438-A allele with triglyceride levels under the additive (β = 0.10, *p* = 0.015), codominant (AA β = 0.37, *p* = 0.011), and recessive models (β = 0.35, *p* = 0.015). Additionally, we corroborate the significant risk association of the rs56371916-C allele for HDL-c levels under additive (β = −0.04, *p* = 0.001), codominant (AA genotype: β = −0.07, *p* = 0.016), and dominant inheritance model (β = −0.06, *p* = 0.0004) (Table S2). We did not find any significant association between SNPs and lipid parameters in the women group (data not shown).

### 2.3. Conditional Analysis

We conditioned the effect of each SNP for each lipid parameter under a dominant model. The conditional analysis showed that the rs2642438 variant on *MARC1* maintained a significant risk association for high triglycerides in the presence of rs6564851 (*p* = 0.014), rs56371916 (*p* = 0.014), or both (*p* = 0.012); these findings were supported by the linear regression analysis. Concerning the rs56371916 SNP on *ADCY5*, it showed a borderline risk association for low HDL-c in the presence of the rs2642438 (*p* = 0.056), rs56371916 (*p* = 0.066), or both (*p* = 0.058); however, the linear regression analysis revealed a significant statistical association (*p* = 4 × 10^−5^, *p* = 6 × 10^−5^, and *p* = 4 × 10^−5^, respectively). In contrast, the rs6564851 SNP on *BCO1* did not show a significant association in the conditional analysis with high triglycerides and low HDL-c (Table S3). 

### 2.4. Association of the Genetic Risk Score with the Lipidic Profile

A genetic risk score (GRS) was constructed for each man, including the *MARC1, ADCY5*, and *BCO1* SNPs (Table 3). We added the rs6564851 SNP on the *BCO1* gene to the GRS model because, recently, we reported a significant association between this variant with the risk for hypertriglyceridemia in a group of middle-aged Mexican adult men [8]. 

For this analysis, we included all the men of the HWCS (Table S4). Hence, we tested several possible GRS model combinations, including two or three variants (rs2642438 on *MARC1*, rs56371916 on *ADCY5,* and rs6564851 on *BCO1*), and questioned their additive effect on the lipid profile. Several GRS models had a significant risk association for high triglycerides and low HDL-c levels (Table 3). However, the GRS model created with the three SNPs showed a significant stepwise increase in triglycerides and a trend for HDL-c levels as a function of the number of risk alleles carried (OR = 2.29, *p* = 0.017; and OR = 1.79, *p* = 0.080; respectively) (Table 3 and Table 4). 

**Figure 1:**
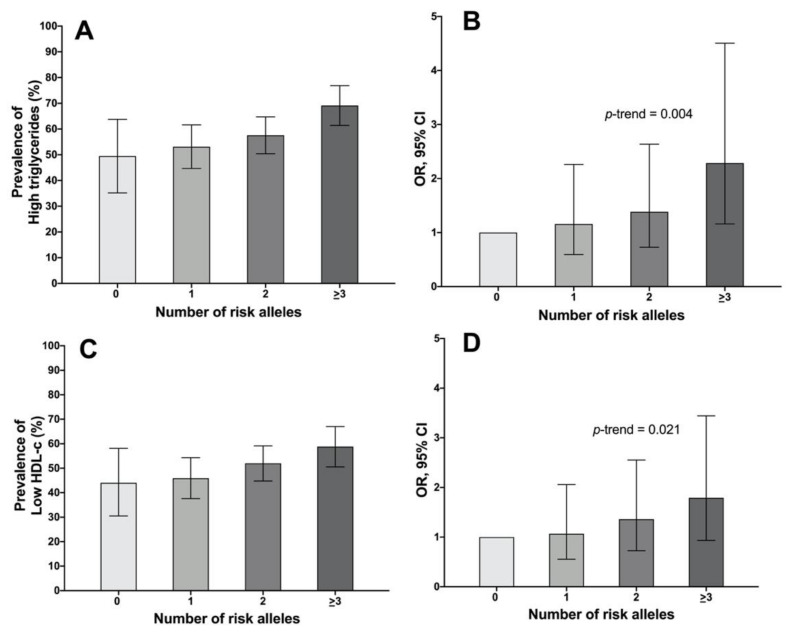
GRS including rs2642438-A *(MARC1)*, rs56371916-C *(ADCY5)*, and rs6564851-A *(BCO1)* for hypertriglyceridemia in Mexican adult men. (**A**) Prevalence of high triglycerides; (**B**) Odd ratio (OR) for high triglycerides (> 3 risk alleles OR = 2.23; 95% CI 1.13–4.42, *p* = 0.022), *p*-trend= 0.004; (**C**) Prevalence of low HDL-c levels; (**D**) OR for low HDL-c (> 3 risk alleles OR = 1.80; 95% CI 0.93–3.46, *p* = 0.079, *p*-trend= 0.021). Models adjusted for age (years), alcohol intake (g/day), smoking status (no, past, current), BMI (normal, overweight, obesity), and lipid-lowering medications (no, yes).

Furthermore, the GRS was associated with an increased prevalence of hypertriglyceridemia and low HDL-c according to the number of risk alleles carried (Figure 1A–D). It is noteworthy that the GRS model with the two *MARC1* and *ADCY5* SNPs was the one that best showed a significant risk association for HDL-c (OR = 3.44, *p* = 0.018) (Table 3).

## 3. Discussion

To the best of our knowledge, this is the first study to analyze the added effect of three polymorphisms rs2642438, rs56371916, and rs6564851 on *MARC1*, *ADCY5*, and *BCO1* genes, respectively, recently associated with lipid metabolism. Interestingly, our results suggest an aggregate effect between the *MARC1*, *ADCY5*, and *BCO1* for hypertriglyceridemia, and the effect appears to be exclusive for men.

Several studies have shown that specific genetic variants are associated with lipid metabolism, and some are significatively relevant in the Mexican population [26]. In this regard, the minor allele frequency (MAF) for the rs2642438-A and rs56371916-C alleles in the HWCS population was 17% and 35%, respectively, slightly lower than reported by the 1000 Genomes Database for the Mexican Ancestry in Los Angeles, California population (26% and 38%, respectively).

Previously, the *MARC1* gene has been associated with protection for all-cause cirrhosis and lower blood hepatic enzyme levels [9,27,28]. In this study, we did not find a significant association with hepatic enzymes (data not shown). This lack of significant association could be due to the average age in the group of men in the HWCS, which was 46.3 years. It has been reported that the incidence of NAFLD occurs mainly in people over 50 years of age [29]. Another possible explanation may be the sample size of men, which may be insufficient to capture the effect of hepatic enzymes in our population.

However, our results showed that the rs2642438-A allele is related to a higher risk for hypertriglyceridemia and a trend for low HDL-c levels only in males. Supporting our findings, a previous GWAS performed on men and women from European cohort populations demonstrated that the *MARC1* variant is related to higher triglycerides and lower HDL-c, LDL-c, and total cholesterol levels [9,13,27].

Although the precise role and physiological function of *MARC1* are unknown, it has been proposed that this lipid phenotype may hint at a possible physiological mechanism by which *MARC1* could participate in NAFLD [12,30]. Accumulating evidence has associated the occurrence of hypertriglyceridemia as a central factor in the progression of the liver, metabolic, and cardiovascular disease [27,31]. Although the specific mechanism by which *MARC1* generates an aggregate effect for hypertriglyceridemia and a trend for low HDL-c levels is unknown, we could speculate on some possible mechanisms. The *MARC1* gene encodes the Mitochondrial Amidoxime-Reducing Component 1, a molybdenum-containing enzyme [9]. This enzyme modulates the nitric oxide bioavailability and L-arginine production, key molecules involved in mitochondrial and endothelial function [10,32]. Experimental studies have shown that hypertriglyceridemia and endothelial dysfunction are closely related through several mechanisms [33]. It has been postulated that endothelial dysfunction is favored by suppressing HDL-c levels, blocking their antiatherogenic action [34]. Therefore, this linking could correlate with a recent study that proposed that the rs2642438 variant confers a deleterious function of the MARC1 protein [11]. Consequently, we could suggest that rs2642438 polymorphism directly or indirectly influences endothelial dysfunction and could generate an imbalance in the triglycerides and HDL-c levels. However, the specific mechanisms remain unknown, and experimental assays must elucidate them. 

Concerning the rs56371916 variant on *ADCY5*, our results showed a significant risk association between the allele-C with low HDL-c and a protective effect for total cholesterol levels only in males. Consistent with our results, Hoffman and colleagues, in a multi-ethnic GWAS, found that the *ADCY5* gene also plays a role in lipid metabolism, mainly associated with HDL-c and total cholesterol plasma concentrations [35]. The possible biological mechanisms underlying these associations could be that the *ADCY5* gene encodes adenylate cyclase 5 protein which mediates G-protein-coupled receptor signaling through the synthesis of cAMP [18]. The literature has documented that cAMP play a pivotal role in modulating apoA1-binding activity and the ATP-binding cassette transporter A1 (ABCA1), promoting the cellular cholesterol efflux, a critical process for the HDL-c circulating levels [36,37,38]. Therefore, we can speculate that the rs56371916 variant on *ADCY5* could modulate the production of cAMP throughout the differential *ADCY5* gene expression, altering the lipid metabolism, total cholesterol, and HDL-c levels [7]. However, further investigations are necessary to corroborate this biological interaction. 

On the other hand, the *BCO1* gene has been involved in lipid metabolism [8,25]. The literature has suggested that the presence of the different rs6564851-G/T alleles could modify the *BCO1* activity by binding several transcription factors [25]. Among them is the putative binding site for the heterodimer peroxisome proliferator-activated receptor-α:retinoid X receptor-α (PPARα:RXRα) in the rs6564851 region. It is one of the most significant regulators of lipid metabolism [39,40]. Activation of PPAR-α leads to a variation in the lipid levels such as triglycerides and HDL-c levels in plasma [41]. Another main transcription factor involved is the intestine-specific homeobox (ISX)-binding site that coincides with the rs6564851 locus. The flanking nucleotide sequence in the promoter region of the scavenger receptor class B type 1 (SR-B1) has been demonstrated [42]. SR-B1 is a key membrane receptor that modulates the HDL-c levels through reverse cholesterol transport, increasing the triglycerides levels in plasma [43,44,45,46,47].

To date, no studies have reported possible sex differences associated with *MARC1* and *ADCY5* genes in humans. However, our findings of the sex-specific association agree with a previous study using the *MARC1* knockout mice model that described a sex-dependent phenotype [30,48]. A recent study using the *ADCY5^-/-^* mice model reported significant differences in the total cholesterol, HDL-c, and LDL-c serum profile between male and female mice, without significant differences reported for triglyceride levels [49]. The authors suggest that *ADCY5* could work as a signaling switch for the apoprotein A1-mediated cholesterol efflux pathway [50]. Furthermore, they concluded that cAMP, produced by *ADCY5*, may regulate cholesterol exocytosis to remove excessive cellular lipids and could increase aromatase expression, a key enzyme involved in estrogen production, promoting distinct phenotypes between males and females [49].

Given differences in triglycerides and HDL-c metabolism between men and women, Salazar and colleagues have reported that the plasma triglycerides/HDL-c concentration ratio varies as a function of gender and racial groups [51,52]. In addition, this study demonstrated that women and men whose triglycerides/HDL-c ratios exceed 2.5 and 3.5, respectively, are significantly more insulin resistant, with a substantially greater cardiovascular risk profile, compared with the rest of the population [51,52].

Using a GRS including *MARC1*, *ADCY5*, and *BCO1* risk alleles, individuals carrying ≥ 3 risk alleles showed a significant stepwise increase in triglycerides and a trend for HDL-c levels as a function of the number of risk alleles. This GRS accounted for 70% of the prevalence of triglycerides in men, while *MARC1*- SNP was the only one that showed a more significant contribution to serum triglyceride levels.

In Mexico, the National Health and Nutrition Survey (2012–2016) has described that females exhibit higher total cholesterol and HDL-c serum concentrations than men, who usually experience a higher risk for hypertriglyceridemia [53]. It has been reported that adult Mexican men usually have a sedentary lifestyle and higher consumption of refined carbohydrates, saturated fat, and sugary and alcoholic drinks, which could be one of the leading causes of dyslipidemia in men [2,54,55,56].

Sexual dimorphism in lipid metabolism seems to result from a complex combination of direct or indirect sex-dependent modulators. Although the specific pathways are unknown, we could speculate on some potential mechanisms. First, there is the differential expression and the effect of insulin sensitivity, adipokines, and genes between men and women [57]. Second, differences in fatty body composition could affect the fatty acid availability for VLDL and triglyceride synthesis [58]. Third, endogenous sex hormones mediate lipid metabolism [59]. For example, testosterone and androgens induce a profile based on reduced HDL-c and ApoA concentrations and increasing LDL-c and triglycerides levels [60]. However, the specific physiological modulators responsible for these lipid differences between sexes remain to be elucidated.

Therefore, this approach might offer the possibility of personalized medicine in men with a specific cardiometabolic profile, characterized by high triglycerides and low HDL-c levels (atherogenic dyslipidemia), which could be assessed, diagnosed, and treated opportunely according to their unique genetic composition and molecular phenotype. Although the GRS can help identify high-risk subgroups for atherogenic dyslipidemia in Mexican men, the specific pathway is unknown, and probably several other polymorphisms in these regions play an important role in the hypertriglyceridemia phenotype in men.

There are several strengths of this study: First, our study reports a GRS associated in a sex-specific manner, while most of the studies propose GRS for the general population independent of sex. Hence, it should be noted that the Mexican population has a differential prevalence of dyslipidemias between men and women [3]. Second, it offers the analysis of a large sample size compared with other observational studies. Third, it provides a rigorous statistical analysis adjusted for potential confounding covariables, decreasing the risk of spurious associations. However, this study has some potential limitations. First, HWCS participants are a select group of health workers located in the central region of Mexico. This may not reflect the health behavior of the entire Mexican population; therefore, the results should be applied with caution to other populations. Second, despite a sufficient overall sample size, the lack of statistical significance for some covariates could reflect low statistical power in the men group (*n* = 579). Third, we cannot rule out population stratification bias in the association analysis because we do not have informative markers of ancestry (AIMs); however, all the HWCS participants included in this study have lived in the central region of Mexico (Cuernavaca, Morelos) for at least three generations. Therefore, we consider that our results would not be affected.

## 4. Materials and Methods

### 4.1. Health Workers Cohort Study

This cross-sectional study included data from 1900 individuals belonging to the HWCS. The HWCS consists of medical, academics, and administrative employees from the Mexican Social Security Institute (IMSS, by its Spanish acronym), located in Cuernavaca, Morelos, Mexico. This Mexican mestizo population-based cohort study focused on the association between genetic and environmental factors in chronic diseases. The cohort design has been previously described in detail in [61]. Data included in the current analysis come from the second sample collection period (2010–2012) and those individuals whose DNA samples and clinical data were available.

This research was performed following the Declaration of Helsinki. The Research and Ethics Committee approved the study protocol and informed consent form from the IMSS (No. 12CEI 09 006 14, 17 May 2016) and the Instituto Nacional de Medicina Genómica (266-17/2016/I, 16 May 2016). Informed consent was obtained from all participants.

### 4.2. Outcome 

Venous blood samples were obtained from each subject for lipids and glucose determination after 8 h of fasting. The concentration of lipids collected was measured once when the participants were enrolled in the study. Triglycerides were measured with a colorimetric method following enzymatic hydrolysis performed with the lipase technique, LDL-c, and HDL-c by the clearance method. All biomedical assays were performed with a Selectra XL instrument (Randox Laboratories Ltd., Antrim, UK), according to the International Federation of Clinical Chemistry and Laboratory Medicine [62]. According to the Adult Treatment Panel-III (ATP-III) criteria, elevated lipid levels were defined as total cholesterol ≥ 200 mg/dL, triglycerides ≥ 150 mg/dL, LDL-c >100 mg/dL, and low HDL-c levels for men < 40 mg/dL and women <50 mg/dL [63].

### 4.3. Genomic DNA Extraction and SNP Genotyping 

Genomic DNA was extracted from peripheral blood using a commercial isolation kit (QIAGEN systems Inc., Valencia, CA), according to the manufacturer’s instructions. The variants rs2642438 (C_1235772_10) on *MARC1* and rs56371916 (C_3035715_20) on *ADCY5* gene were genotyped. The genotypes of *n* = 1441 individuals of the rs6564851 on the *BCO1* gene have been previously reported [8]. The remaining samples (*n* = 459) from HWCS were genotyped for this study with the assay C_28949771_10. Genotyping was performed using predesigned TaqMan SNP Genotyping assays (Applied Biosystems, Massachusetts, MA, USA) in a QuantStudio 7 Flex Real-Time PCR system (Applied Biosystems, Massachusetts, MA, USA). The automatic variant call was carried out with the SDS software version 2.2.1.

### 4.4. Construction of the Genetic Risk Score

The GRS was constructed from three SNPs located on *MARC1* (rs2642438), *ADCY5* (rs56371916), and *BCO1* (rs6564851) genes, which were selected based on a primary risk association for high triglycerides levels in the study population. The GRS was estimated by adding the number of risk alleles from these SNPs in everyone (0 for homozygotes for the non-risk allele, 1 for heterozygotes, and 2 for homozygotes for the risk allele). The reference group was considered by individuals not carrying any risk alleles.

### 4.5. Covariates

Demographic, lifestyle (such as physical activity, smoking status, and diet), and clinical data were evaluated by self-administered questionnaires. Dietary data were collected using a 116-item semiquantitative food frequency questionary (FFQ); its validity and reliability have been previously reported [61,64]. The reported frequency for each food item was converted to a daily intake. Food composition tables compiled by the National Institute of Public Health were used to determine the nutrient compositions of all foods [64]. BMI was determined (calculated as weight (kg)/height (m)^2^) and classified into three groups: normal < 25 kg/m^2^, overweight 25–29.9 kg/m^2^, and obesity ≥ 30 kg/m^2^. We calculated leisure-time physical activity with data from a previously validated questionnaire [62] and classified participants as inactive (<150 min/week of moderate to vigorous activity) or active (≥150 min/week of moderate to vigorous activity) according to the World Health Organization (WHO) criteria. Liver enzymes ALT and AST were measured using a commercial test and were considered normal (<40 international units per liter (UI/L)).

### 4.6. Statistical Analyses

A descriptive analysis of all variables was stratified by sex and genotype of the rs2642438 and the rs56371916 SNPs. The continuous sociodemographic and biochemical characteristics are presented as medians and interquartile range (25–75 percentile). The categorical variables were presented as percentages. To investigate differences in participant characteristics, we compared continuous variables using the *Dunn test* and categorical variables using the chi-square test. Natural logarithmic transformation (Ln) was applied for lipids measures. We evaluated the association between the three SNPs and each log-transformed lipidic parameter (triglycerides, HDL-c, LDL-c, and total cholesterol) using linear models. To estimate the association between SNPs and dyslipidemias, we computed adjusted OR and 95% CI with a logistic regression model adjusted for variables such as age, sex, BMI category, physical activity, alcohol consumption, and smoking status. Finally, we evaluated the association between GRS and lipids using a linear and logistic regression model. All statistical analyses were performed using the STATA software version 14.0 (StataCorp LP, College Station, TX, USA). A *p*-value ≤ 0.05 was designated as the cutoff for statistical significance.

## 5. Conclusions

In conclusion, our study suggests a risk effect between the variants rs2642438 on *MARC1* and rs56371916 on *ADCY5* for hypertriglyceridemia, with sex-specific effects. The GRS based on the three genetic variants of *MARC1, ADCY5*, and *BCO1* has an additive risk effect for hypertriglyceridemia and suggest atherogenic dyslipidemia based on high triglycerides and low HDL-c levels in adult Mexican men. The current finding adds insight into the role of these loci in lipid metabolism. However, additional studies in different populations are needed to determine if the observed association has a clinical relevance according to sex.

## Figures and Tables

**Table 1:** Clinical characteristics of the individuals belonging to the Health Workers Cohort Study.

	Men = 579	Women = 1321	*p*
Age ^a^, (years)	46.3 (14.6)	52.3 (14.9)	<0.001
BMI ^a^, (kg/m^2^)	26.6 (24.3–29.2)	26.9 (24.1–30.3)	0.115
Overweight, %	48.7	40.4	0.0008
Obesity, %	19.9	26.3	0.0028
Leisure time physical activity ^a^ (hour/week)	1.7 (0.4–5)	1.1 (0.2–3.5)	<0.001
Active (>150 min/week), %	42.3	31.1	<0.001
Smoking status, %			
Current, %	20.9	8.9	<0.001
Past, %	39.2	22.5	<0.001
ALT ^a^, (U/L)	25 (19–35)	20 (15–29)	<0.001
AST ^a^, (U/L)	25 (21–31)	23 (20–30)	0.0001
Serum total cholesterol ^a^, (mg/dL)	192 (168–222)	199 (172–226)	0.0003
High total cholesterol ^b^, %	40.6	48.8	0.0008
Serum HDL-c ^a^, (mg/dL)	39 (34–46)	46 (39–54)	<0.001
Low HDL-c ^c^, %	51.8	64.0	<0.001
Serum LDL-c ^a^ (mg/dL)	115 (96–144)	121 (99–146)	0.007
High LDL-c ^d^, %	70.5	74.2	0.094
Serum triglycerides ^a^, (mg/dL)	168 (119–245)	150 (109–201)	<0.0001
High triglycerides ^e^, %	58.4	50.4	0.001
Lipid-lowering treatment, %	11.5	13.9	0.154
Diet			
Energy intake ^a^ (kcal/day)	1936 (1457–2549)	1687 (1242–2221)	<0.001
Carbohydrate ^a^ (% energy)	64.6 (58.1–70.5)	66.5 (60.6–71.8)	<0.001
Protein ^a^ (% energy)	12.3 (10.6–14.1)	12.5 (11.0–14.3)	0.061
MUFAs ^a^ (% energy)	8.4 (6.8–10.5)	8.6 (7.0–10.4)	0.210
PUFAs ^a^ (% energy)	1.8 (1.5–2.2)	1.9 (1.6–2.2)	0.199
Alcohol ^a^ (g/day)	2.8 (0.6–7.5)	0.6 (0–1.8)	<0.001

Abbreviations: BMI—Body Mass Index; HDL-c—high-density lipoprotein-cholesterol; LDL-c—low-density lipoprotein-cholesterol; ALT—Alanine aminotransferase; AST—Aspartate aminotransferase; MUFAs—Monounsaturated fatty acids; PUFAs—Polyunsaturated fatty acids. Wilcoxon rank-sum test was used for continuous variables, and a 2-sample proportion test by categorical variables. *p* < 0.05 was considered statistically significant. ^a^ Median (P25-P75); ^b^ High total cholesterol ≥ 200 mg/dL; ^c^ Low HDL-c ≤40 mg/dL for men and ≤50 mg/dL for women; ^d^ High LDL-c ≥ 100 mg/dL and ^e^ High triglycerides ≥ 150 mg/dL.

**Table 2:** Association between rs2642438 on *MARC1* and rs56371916 on *ADCY5* with lipid profile in men from HWCS.

	rs2642438 *MARC1*					rs56371916 *ADCY5*			
Model	High Total Cholesterol ^a^ OR (95% CI)	Low HDL-c ^b^ OR (95% CI)	High TG ^c^ OR (95% CI)	High LDL-c ^d^ OR (95% CI)	Model	High Total Cholesterol ^a^ OR (95% CI)	Low HDL-c ^b^ OR (95% CI)	High TG ^c^ OR (95% CI)	High LDL-c ^d^ OR (95% CI)
Additive									
	0.86 (0.62–1.21)	1.26 (0.90–1.75)	1.57 (1.10–2.24)	1.37 (0.94–1.98)		0.93 (0.73–1.19)	1.27 (0.99–1.63)	1.03 (0.80–1.33)	0.94 (0.72–1.23)
*p*	0.390	0.181	0.013	0.101	*p*	0.569	0.060	0.830	0.647
Codominant									
GG *					TT *				
GA	0.83 (0.57–1.23)	1.24 (0.85–1.83)	1.44 (0.96–2.14)	1.41 (0.92–2.15)	TC	0.85 (0.59–1.23)	1.26 (0.88–1.81)	1.27 (0.87–1.86)	0.74 (0.50–1.10)
*p*	0.357	0.263	0.075	0.114	*p*	0.396	0.211	0.209	0.140
AA	0.89 (0.28–2.84)	1.65 (0.51–5.31)	4.58 (0.95–22.03)	1.57 (0.42–5.89)	CC	0.93 (0.54–1.59)	1.62 (0.94–2.79)	0.90 (0.52–1.55)	1.07 (0.58–1.95)
*p*	0.838	0.263	0.057	0.503	*p*	0.784	0.082	0.694	0.834
Recessive									
GG + GA *					TT + TC *				
AA	0.93 (0.29–2.96)	1.55 (0.48–4.98)	4.16 (0.87–19.9)	1.44 (0.38–5.36)	CC	1.00 (0.60–1.67)	1.45 (0.87–2.41)	0.80 (0.47–1.34)	1.24 (0.70–2.18)
*p*	0.903	0.461	0.075	0.590	*p*	0.993	0.159	0.387	0.457
Dominant									
GG *					TT *				
GA + AA	0.84 (0.58–1.22)	1.27 (0.88–1.85)	1.54 (1.04–2.28)	1.42 (0.94–2.14)	TC + CC	0.87 (0.62–1.23)	1.33 (0.95–1.88)	1.17 (0.82–1.67)	0.81 (0.55–1.17)
*p*	0.355	0.207	0.030	0.096	*p*	0.427	0.097	0.377	0.255

Models adjusted for age (years), alcohol intake (g/day), BMI (normal, overweight, and obesity), lipid-lowering treatment (no, yes), physical activity (< 30 min/day), and smoking (never, past, current). ^a^ High total cholesterol ≥ 200 mg/dL; ^b^ Low HDL-c ≤ 40 mg/dL for men and ≤ 50 mg/dL for women; ^c^ High LDL-c ≥ 100 mg/dL and ^d^ High triglycerides ≥ 150 mg/dL. Triglycerides (TG). * Genotype of reference.

**Table 3:** Construction of the genetic risk score associated with lipid parameters in Mexican adult men.

	Number of Risk Alleles		Low HDL-c		High Triglycerides		High LDL-c		High Total Cholesterol	
Model SNP/Gene		*n* (%)	OR (IC 95%)	*p*	OR (IC 95%)	*p*	OR (IC 95%)	*p*	OR (IC 95%)	*p*
rs2642438 *MARC1* rs6564851 *BCO1*	0 *	119(20.5)								
	1	262(45.1)	1.34 (0.85–2.12)	0.205	1.03 (0.65–1.65)	0.890	0.86 (0.53–1.40)	0.543	0.71 (0.45–1.12)	0.143
	2	161(27.7)	1.83 (1.11–3.02)	0.018	1.69 (1.00–2.85)	0.048	1.15 (0.67–1.97)	0.616	0.85 (0.52–1.39)	0.520
	≥3	39(6.7)	0.71 (0.32–1.58)	0.408	3.83 (1.55–10.10)	0.005	1.49 (0.60–3.68)	0.385	0.71 (0.32–1.58)	0.398
rs2642438 *MARC1* rs56371916 *ADCY5*	0 *	552(29.1)								
	1	812(42.7)	1.40 (0.93–2.12)	0.105	1.35 (0.89–2.05)	0.162	0.77 (0.50–1.20)	0.249	0.87 (0.58–1.31)	0.504
	2	446(23.5)	1.42 (0.87–2.31)	0.162	1.39 (0.84–2.30)	0.195	1.28 (0.74–2.22)	0.382	0.78 (0.48–1.27)	0.321
	≥3	90(4.7)	3.46 (1.24–9.64)	0.018	1.83 (0.68–4.88)	0.229	1.38 (0.48–4.04)	0.551	0.92 (0.36–2.33)	0.865
rs56371916 *ADCY5* rs6564851 *BCO1*	0 *	80(13.8)								
	1	195(33.5)	0.91 (0.52–1.58)	0.733	1.11 (0.63–1.95)	0.723	0.83 (0.45–1.52)	0.546	0.98 (0.56–1.70)	0.941
	2	213(36.6)	1.15 (0.67–1.98)	0.617	1.39 (0.79–2.42)	0.252	0.83 (0.46–1.51)	0.544	0.87 (0.50–1.49)	0.603
	≥3	94(16.2)	1.61 (0.85–3.06)	0.147	1.80 (0.93–3.50)	0.082	0.94 (0.47–1.89)	0.870	0.91 (0.48–1.71)	0.760
rs2642438 *MARC1* rs56371916 *ADCY5* rs6564851 *BCO1*	0 *	53(9.2)								
	1	160(27.6)	1.08 (0.56–2.08)	0.823	1.16 (0.59–2.28)	0.662	0.59 (0.28–1.24)	0.164	0.73 (0.38–1.40)	0.347
	2	207(35.8)	1.37 (0.73–2.58)	0.326	1.32 (0.69–2.54)	0.397	0.70 (0.34–1.44)	0.335	0.68 (0.36–1.32)	0.223
	≥3	159(27.5)	1.80 (0.93–3.46)	0.079	2.23 (1.13–4.42)	0.022	0.93 (0.44–1.98)	0.858	0.69 (0.36–1.32)	0.258

Models adjusted for age (years), alcohol intake (g/day), BMI (normal, overweight, and obesity), lipid-lowering treatment (no, yes), physical activity (<30 min/day, ≥30 min/day), smoking (never, past, current), energy intake, carbohydrate intake, and fat intake. * Reference group.

**Table 4:** Comparison of the lipid profile according to the number of risk alleles in Mexican adult men.

	Number of Risk Alleles (rs2642438-A *MARC1*, rs56371916-C *ADCY5*, rs6564851-A *BCO1)*				
Characteristic	0 *	1	2	≥3	*p*
	*n* = 53 (9.2%)	*n* = 160 (27.5%)	*n* = 207 (35.8%)	*n* = 159 (27.5%)	(0 vs. ≥ 3)
Triglycerides, mg/dL ^a^	153 (115–234)	156 (114–241)	171 (120–249)	171 (129–253)	0.086
High triglycerides, % ^b^	52.8	53.1	57.5	66.7	0.069
HDL-c, mg/dL ^a^	41 (35.4–49.1)	41 (35–47)	39 (34–45.4)	37.7 (33.6–44)	0.004
Low HDL-c, % ^c^	45.3	45.6	52.7	59.1	0.079
Total cholesterol, mg/dL ^a^	199 (177–229)	192 (167–224)	190 (166–221)	192 (168–217)	0.073
High total cholesterol, % ^d^	49.1	41.3	38.7	39.6	0.225
LDL-c, mg/dL ^a^	112 (100–146)	114 (95–147)	115 (96–140)	116 (98–142)	0.406
High LDL-c, % ^e^	76.5	67.5	69	73.6	0.675

Abbreviations: HDL-c—High-density lipoprotein-cholesterol; LDL-c—low-density lipoprotein-cholesterol. *Dunn’s* test was used for continuous variables and a 2-sample proportion test by categorical variables. *P* < 0.05 was considered statistically significant. ^a^ Median (P25-P75); ^b^ High triglycerides ≥ 150 mg/dL; ^c^ Low HDL-c ≤ 40 mg/dL for men and ≤ 50 mg/dL for women; ^d^ High total cholesterol ≥ 200 mg/dL; ^e^ High LDL-c ≥ 100 mg/dL. * Reference group.

## Data Availability

The datasets analyzed in this study are available from the corresponding author upon reasonable request.

## Supplementary Materials

The following supporting information can be downloaded at: https://www.mdpi.com/article/10.3390/ijms231911815/s1.

## Abbreviations

ABCA1—ATP-binding cassette transporter A1; ADCY5—Adenylate cyclase 5; ALT—Alanine aminotransferase; AST—Aspartate aminotransferase; ATP-III—Adult Treatment Panel-III; BCO1—β-Carotene-15,15′-oxygenase; BMI—Body Mass Index; cAMP—cyclic AMP; CI: Confidence interval; CVD—Cardiovascular disease; FFQ—Food frequency questionary; GRS—Genetic risk score; GWAS—Genome-wide association studies; HDL-c—High-density lipoprotein cholesterol; HWCS—Health Workers Cohort Study; ISX—Intestine-specific homeobox; LDL-c—Low-density lipoprotein cholesterol; MARC1—Mitochondrial Amidoxime Reducing Component 1; MAF—Minor allele frequency; MetS—Metabolic syndrome; MUFAs—Monounsaturated fat; NAFLD—Nonalcoholic fatty liver disease; OR—Odd ratio; PPARα—Peroxisome proliferator-activated receptor-α; PUFAs—Polyunsaturated fat; RXRα—Retinoid X receptor-α; SNP—Single nucleotide polymorphism; SR-B1—Scavenger receptor class B type 1; SREBP1—Sterol regulatory-element-binding protein 1; T2D—Type 2 diabetes; WHO—World Health Organization.
