# Supplementary material for: Association of MARC1, ADCY5, and BCO1 Variants with the Lipid Profile, Suggests an Additive Effect for Hypertriglyceridemia in Mexican Adult Men
Source: Int J Mol Sci. 2022 Oct 5;23(19):11815. doi: 10.3390/ijms231911815 (PMC9569691; doi:10.3390/ijms231911815)
Supplement: Supplementary file 1 [file ijms-23-11815-s001.zip › ijms-1932086-supplementary.pdf]

Table S1. Characteristics of the study population categorized by sex and genotypes of the SNPs.

	rs2642438 *MARC1*						rs56371916 *ADCY5*					
	**Men, *n*=592**			**Women, *n*=1,357**			**Men, *n*=583**			**Women, *n*=1,344**		
	**GG *n*=414**	**GA+AA *n*=178**	***P****	**GG *n*=903**	**GA+AA *n*=454**	***P****	**TT *n*=259**	**TC+CC *n*=324**	***P****	**TT *n*=573**	**TC+CC *n*=771**	***P****
Age [a], (years)	46 (36-57)	46 (36-56)	0.715	54 (43-63)	53 (42-63)	0.984	46 (37-57)	46 (35-56)	0.522	53 (42-63)	54 (43-63)	0.261
BMI [a], (kg/m^2)	26.5 (24.2-29.3)	26.6 (24.6-28.7)	0.795	26.9 (24-30.1)	26.7 (24.1-30.1)	0.706	26.7 (24.3-28.9)	26.5 (24.1-29.2)	0.929	29.9 (23.9-30.4)	26.8 (24.1-29.9)	0.861
Overweight, %	46.9	53.4	0.147	40.1	41.6	0.600	51.4	47.2	0.313	37.9	42.5	0.089
Obesity, %	20.5	18.0	0.484	26.0	26.0	1.000	18.2	20.4	0.504	27.1	24.9	0.362
ALT [a], (U/L)	25 (19-34)	25 (21-31)	0.979	21 (15-29)	20 (15-28)	0.544	25 (19-34)	25 (19-35)	0.794	20 (15-27)	20 (16-29)	0.301
AST [a], (U/L)	24 (19-34)	25 (21-32)	0.688	24 (20-30)	23 (19-29)	0.123	24 (21-31)	26 (21-32)	0.324	23 (19-29)	24 (20-30)	0.116
Total cholesterol [a], (mg/dL)	191 (166-223)	192 (171-217)	0.718	200 (175-231)	196 (170-223)	0.032	194 (172-227)	187 (164-217)	0.011	199 (174-227)	198 (171-227)	0.885
HDL-c [a], (mg/dL)	40 (34-46)	39 (34-44)	0.127	46 (39-54)	46 (39-54)	0.836	40.2 (35-47)	38.8 (33.7-44.4)	0.002	45.3 (39.7-53.8)	46.1 (39-54)	0.815
LDL-c [a], (mg/dL)	115 (95-145)	116 (100-138)	0.875	122 (100-149)	119 (99-143)	0.143	117 (98-144)	115 (94-143)	0.273	121 (100-146)	121 (99-147)	0.849
Triglycerides [a], (mg/dL)	164 (116-234)	177 (128-277)	0.053	152 (110-204)	149 (107-197)	0.370	169 (115-250)	168 (123-240)	0.551	149 (108-203)	152 (109-199)	0.808
High Triglycerides [b], %	55.6	65.7	0.028	50.9	49.6	0.651	56.8	60	0.435	49.4	51.1	0.537
Energy intake [a], (kcal/day)	1934 (1472-2508)	1987 (1400-2592)	0.913	1698 (1270-2212)	1671 (1218-2266)	0.435	2048 (1548-2658)	1900 (1442-2439)	0.060	1698 (1272-2278)	1676 (1235-2174)	0.258

Abbreviations: Body mass index (BMI); high-density lipoprotein-cholesterol (HDL-c); low-density lipoprotein-cholesterol (LDL-c); Alanine aminotransferase (ALT); Aspartate aminotransferase (AST); Monounsaturated fatty acids (MUFAs); Polyunsaturated fatty acids (PUFAs).

* *P* values were calculated using the Wilcoxon rank-sum test by continuous variables and 2-sample proportion test by categorical variables. *P* value: GG vs GA+AA; $P < 0.05$ was considered statistically significant. [a] Median (P25-P75); [b] High triglycerides ≥ 150 mg/dL.

Table S2. Association between rs2642438 and rs56371916 with lipid profile in men.

	rs2642438 *MARC1*					rs56371916 *ADCY5*			
**Model**	**Total cholesterol β (95% CI)**	**HDL-c β (95% CI)**	**Triglycerides β (95% CI)**	**LDL-c β (95% CI)**	**Model**	**Total cholesterol β (95% CI)**	**HDL-c β (95% CI)**	**Triglycerides β (95% CI)**	**LDL-c β (95% CI)**
Additive									
	0.005 (-0.03,0.04)	-0.03 (-0.07,0.003)	0.10 (0.02,0.18)	0.002 (-0.04,0.05)		-0.03 (-0.05,-0.004)	-0.04 (-0.07,0.02)	-0.002 (-0.06,0.06)	-0.01 (-0.04,0.02)
P	0.779	0.074	0.015	0.941	*P*	0.022	0.001	0.958	0.558
Codominant									
GG*					TT*				
GA	-0.003 (-0.04,0.032)	-0.03 (-0.07,0.008)	0.07 (-0.03,0.16)	-0.0006 (-0.052,0.051)	TC	-0.05 (-0.08,-0.01)	-0.06 (-0.10,-0.03)	0.05 (-0.04,0.14)	-0.04 (-0.09,0.01)
P	0.846	0.121	0.160	0.981	*P*	0.010	0.001	0.323	0.091
AA	0.05 (-0.06,0.16)	-0.06 (-0.18,0.06)	0.37 (0.08,0.65)	0.01 (-0.14,0.17)	CC	-0.04 (-0.09,0.01)	-0.07 (-0.13,-0.01)	-0.04 (-0.17,0.09)	0.01 (-0.07,0.08)
P	0.378	0.309	0.011	0.854	P	0.119	0.016	0.549	0.867
Recessive									
GG+GA					TT+TC				
AA	0.05 (-0.06,0.16)	-0.05 (-0.17,0.07)	0.35 (0.07,0.63)	0.01 (-0.14,0.17)	CC	-0.02 (-0.07,0.03)	-0.04 (-0.09,0.02)	-0.06 (0.19,0.06)	0.03 (-0.04,0.10)
P	0.366	0.379	0.015	0.852	*P*	0.463	0.162	0.325	0.444
Dominant									
GG*					TT*				
GA+AA	0.0004 (-0.04,0.04)	-0.03 (-0.07,0.004)	0.09 (-0.001,0.18)	0.0006 (-0.05,0.05)	TC+CC	-0.04 (-0.08,-0.01)	-0.06 (-0.10,-0.03)	0.03 (-0.06,0.11)	-0.03 (-0.08,0.01)
P	0.982	0.086	0.054	0.983	*P*	0.007	0.0004	0.555	0.185

Models adjusted for age, body mass index, lipid-lowering treatment, physical activity, smoking, energy intake. *Reference. [a] High total cholesterol ≥ 200 mg/dL; [b] Low HDL-c ≤40 mg/dL for men and ≤50 mg/dL for women; [c] High LDL-c ≥ 100 mg/dL and [d] High triglycerides ≥ 150 mg/dL. Lipids were natural logarithm transformed.

Table S3. Conditional analysis between the three polymorphisms with lipids levels in men.

SNP/Gene	Conditioned by	High triglycerides		Triglycerides levels*		Low HDL-c		HDL-c levels*	
		OR (95% CI)	*P*	β (95% CI)	*P*	OR (95% CI)	*P*	β (95% CI)	*P*
rs2642438 *MARC1*	rs6564851	1.65 (1.11-2.46)	0.014	0.10 (0.004,0.19)	0.041	1.18 (0.81-1.73)	0.376	-0.03 (-0.07,0.01)	0.153
	rs56371916	1.65 (1.11-2.47)	0.014	0.10 (0.004,0.19)	0.041	1.21 (0.83-1.77)	0.322	-0.03 (-0.07,0.006)	0.094
	rs6564851 and rs56371916	1.68 (1.12-2.50)	0.012	0.10 (0.006,0.19)	0.038	1.22 (0.83-1.78)	0.313	-0.03 (-0.07,0.005)	0.091
rs56371916 *ADCY5*	rs2642438	1.22 (0.85-1.74)	0.288	0.03 (-0.06,0.11)	0.535	1.41 (0.99-1.99)	0.056	-0.08 (-0.11,-0.04)	$4x10^{-5}$
	rs6564851	1.18 (0.82-1.68)	0.376	0.02 (-0.06,0.11)	0.634	1.39 (0.98-1.01)	0.066	-0.07 (-0.11,-0.04)	$6x10^{-5}$
	rs2642438 and rs6564851	1.21 (0.84-1.74)	0.297	0.03 (-0.06,0.11)	0.549	1.40 (0.98-1.99)	0.058	-0.08 (-0.11,-0.04)	$4x10^{-5}$
rs6564851 *BCO1*	rs2642438	1.41 (0.96-2.07)	0.082	0.05 (-0.04,0.14)	0.250	1.18 (0.81-1.71)	0.383	-0.02 (-0.06,0.02)	0.422
	rs56371916	1.38 (0.94-2.02)	0.101	0.05 (-0.04,0.14)	0.203	1.17 (0.80-1.70)	0.415	-0.01 (-0.05,0.03)	0.494
	rs2642438 and rs56371916	1.40 (0.96-2.06)	0.084	0.05 (-0.04,0.14)	0.255	1.17 (0.81-1.70)	0.403	-0.01 (-0.05,0.02)	0.463

Dominant model adjusted for age (years), body mass index (kg/m^2), physical activity (inactive/active), lipid-lowering medications (no, yes), smoking (no, current, past); OR: Odd ratio, CI: confidence interval. [a] High triglycerides ≥ 150 mg/dL; [b] Low HDL-c ≤40 mg/dL for men and ≤50 mg/dL for women. *P* < 0.05 was considered statistically significant. Lipids were natural logarithm transformed.

Table S4. Association between rs6564851 on *BCO1* with lipid profile in men belonging to the HWCS.

	rs6564851 *BCO1*			
**Model**	**High Total cholesterol [a] OR (95% CI)**	**Low HDL-c [b] OR (95% CI)**	**High Triglycerides [c] OR (95% CI)**	**High LDL-c [d] OR (95% CI)**
Additive				
	0.96 (0.75-1.23)	1.06 (0.83-1.37)	1.33 (1.02-1.72)	0.99 (0.76-1.29)
P	0.726	0.623	0.031	0.947
Codominant				
GG*				
GA	1.01 (0.68-1.50)	1.21 (0.82-1.79)	1.25 (0.83-1.87)	0.94 (0.61-1.43)
P	0.967	0.344	0.278	0.760
AA	0.90 (0.54-1.50)	1.09 (0.66-1.81)	1.81 (1.06-3.07)	1.00 (0.58-1.72)
P	0.683	0.743	0.029	0.996
Recessive				
GG+GA*				
AA	0.89 (0.57-1.40)	0.97 (0.62-1.51)	1.58 (0.99-2.52)	1.04 (0.65-1.67)
P	0.627	0.889	0.058	0.872
Dominant				
GG*				
GA+AA	0.98 (0.67-1.42)	1.18 (0.81-1.71)	1.38 (0.94-2.02)	0.95 (0.64-1.42)
P	0.906	0.514	0.099	0.813

Models adjusted for age, body mass index, lipid-lowering treatment, physical activity, smoking, energy intake. *Genotype of reference. [a] High total cholesterol ≥ 200 mg/dL; [b] Low HDL-c ≤40 mg/dL for men and ≤50 mg/dL for women; [c] High LDL-c ≥ 100 mg/dL and [d] High triglycerides ≥ 150 mg/dL.
